# Cyclopia: the one-eyed fetus

**DOI:** 10.11604/pamj.2017.26.156.11275

**Published:** 2017-03-15

**Authors:** Faten Limaiem, Saadia Bouraoui

**Affiliations:** 1 University of Tunis El Manar, Tunis Faculty of Medicine, 1007 Tunisia; 2 University Hospital Mongi Slim, La Marsa, Sidi Daoued, 2046 Tunisia

**Keywords:** Cyclopia, fetus, holoprosencephaly

## Image in medicine

A 36 year-old female patient gravida 2 para 1, with no particular past medical history consulted her gynecologist at 14 weeks' gestation for pelvic pain. Her first pregnancy was uneventful and she did not take any specific drugs prior or during her pregnancy. Consanguinity and hereditary diseases were denied. Ultrasonography revealed a retention image measuring 67,2 mm with no cardiac pulsations (A). A male cyclopean fetus was delivered by induced labour. The fetus weighed 13,5 g and the placenta 45,4 g. On examination, the fetus was monophtalmic with completely fused eyes (B). In the face was a central proboscis located above the single orbital cavity (B). It had a rhombic form presenting with two lateral commissures and one caruncle at the inferior point of union. There was one lacrimal punctum in the midline and no median cleft. The nasal structures were absent. The upper lip looked normal with both, philtrum and tuberculum labii superioris. The lower lip and the mandible were normal. The brain and the rest of the organs were macerated. The examination of the placenta did not disclose any anomaly. Genetic analysis was not performed.

**Figure 1:**
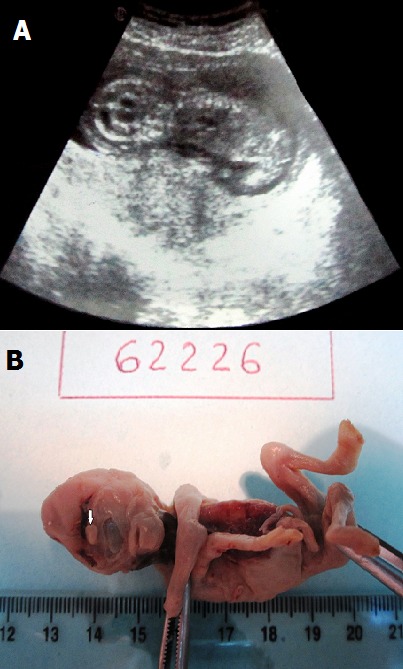
(A) ultrasonography revealed a retention image measuring 67,2 mm with no cardiac pulsations; (B) the fetus was monophtalmic with completely fused eyes. In the face, there was a central proboscis (white arrow) located above the single orbital cavity. The nasal structures were absenT

